# Assessing depression and anxiety among young adults after epidemics and pandemics: a cross-sectional study in Anyang, China

**DOI:** 10.1038/s41598-024-53292-w

**Published:** 2024-02-02

**Authors:** Guoqing Wang, Kamal Sabran

**Affiliations:** Department of New Media Design and Technology, School of the Arts, Universiti Sains Malaysia, 11800 Gelugor, Penang Malaysia

**Keywords:** Psychology, Human behaviour

## Abstract

It has been well established that pandemics affect mental health, yet few studies have been conducted in China regarding this issue following COVID-19's gradual decline and the recent H1N1 influenza outbreak. In response to this research gap, this investigation explores the risk factors linked to depression and anxiety symptoms among young adults in this specific setting. Data were collected via an online cross-sectional survey of 385 young adults living in Anyang city, Henan Province, China, between June 15 and July 21, 2023. Respondents were assessed for anxiety and depression symptoms using the GAD-7 and PHQ-9 scales. Additionally, to examine the factors that influenced the study, we utilized an ordered logit regression model. Results revealed depression and anxiety prevalence rates of 33.3% and 21.6%, respectively. Several factors were found to increase the likelihood of depression and anxiety among young adults, including gender, age, education status, marital status, and attitudes towards epidemics. Participants' concerns about pandemics and viruses had a significant negative impact relationship on depression levels. Women report moderate to severe anxiety more frequently than men. An evident correlation can be observed between the educational attainment level and the influence of depression and anxiety.

## Introduction

The global landscape has witnessed a succession of significant epidemics, some attaining pandemic status, such as COVID-19, Middle East respiratory syndrome (MERS), influenza A (H1N1), Severe Acute Respiratory Syndrome (SARS), and HIV/AIDS^1^. Notably, major epidemics like MERS, influenza A (H1N1), SARS and COVID-19 have left enduring and widespread impacts on mental health^2–5^. The COVID-19 pandemic, in particular, has escalated anxiety and depression levels within the Chinese population^6^.

COVID-19 was cleared of preventative and control measures, as outlined in Chinese Law on the Prevention and Control of Infectious Diseases, on January 8, 2023^7^. However, just as the threat of COVID-19 was easing, a resurgence of H1N1 influenza in various Chinese cities, including Zhengzhou, emerged in March 2023^8^. Considering this resurgence, the mental health of the Chinese population faces a grave threat.

Zhengzhou, Henan province, which detected 19 cases of imported COVID-19 on January 21, 2020, faced a significant challenge. By January 9, 2023, it was reported that 89 percent of the province's nearly 100 million population had been infected^9^. Henan province recorded the highest cumulative number of deaths after Hubei and Shanghai^10^. Notably, Anyang City, accounting for a significant percentage of the province’s confirmed cases^11^. The prolonged isolation policy implemented in Henan after the COVID-19 outbreak resulted in a multifaceted problem concerning young adults' mental health^12^.

On March 14, 2023, influenza A subtype A H1N1 dominated in Anyang City, as indicated by a surveillance report from the National Influenza Centre^13^. The rising rate of test-positive influenza cases in southern and northern provinces underscored the increasing prevalence of H1N1^14^. With distinct symptoms and recommendations for control, the coexistence of COVID-19 and H1N1 placed substantial strain on the mental health of Anyang City's residents.

In general, patients with COVID-19 were advised to isolate, exacerbating psychological distress, particularly among the younger population^15^. Heightened concerns about young people's mental health have been amplified by the cumulative impact of prolonged isolation, an uncertain future, and job insecurity^15^. In previous studies, such as a multi-country systematic review during the COVID-19 pandemic, young people have been emphasized as vulnerable to psychological distress^16^. Similarly, research on H1N1 in Guangzhou highlighted misunderstandings and elevated psychological distress among college students^17^.

Against this backdrop, our study aims to investigate post-pandemic mental health, specifically depression and anxiety, among young people living in Anyang, Henan Province, China, as well as the factors associated with them. As existing research primarily addressed COVID-19's enduring effects, the current study extends its scope beyond, presenting a unique scenario of a dual public health crisis. Moreover, our investigation intends to provide valuable insights into the distinctive circumstances that unfold after pandemics, shedding light on the potential compounding effects of successive infectious disease emergencies. In navigating the intricate landscape of public health challenges, this nuanced exploration is indispensable for a comprehensive understanding of the dynamic and multifaceted nature of mental health.

## Methods

### Study design

In order to conduct this research, we conducted a cross-sectional survey of young adults living in Anyang from June 15 to July 21, 2023.

### Setting and participants

Participants were selected using predetermined criteria. Those who qualified for inclusion must be aged 18–39 years, live within the geographic boundaries of Anyang City, express willingness to participate, and provide informed consent. During this stage of life, an individual's financial stability, physical health, and overall well-being are all greatly influenced^18,19^. Exclusion criteria include individuals outside the specified age range, individuals living outside Anyang, individuals who do not wish to participate, and individuals with severe cognitive impairments that prevent them from accurately answering the survey. Tencent Questionnaire, which is one of the largest survey companies in China^20^, was used to distribute the survey to the target population in the selected study site. Respondents were selected using stratified random sampling based on gender, age, and education^21^. The Tencent questionnaire platform was used to distribute a total of 385 questionnaires.

### Instruments

The levels of depression among respondents were assessed using the Patient Health Questionnaire-9 (PHQ-9), a novel instrument employed for diagnosing primary care's prevalent conditions such as depression and other mental health^22^. According to the DSM-IV criteria, the PHQ-9 depression module scores nine conditions using a scale ranging from 0 to 3 (0 = not at all, 1 = several days, 2 = more than half the time, 3 = nearly every day)^22^. In the PHQ-9 summative scores, a variety of depression levels are noted: 5 (mild), 10 (moderate), 15 (moderately severe), and 20 (severe)^22^. According to Costantini et al., a score of 10 is an appropriate threshold for assessing the burden of depressive symptoms^23^. The internal consistency and reliability of PHQ-9 were found to be outstanding in this study, as indicated by a high Cronbach's alpha coefficient of 0.908.

GAD-7 (Generalized Anxiety Disorder Scale-7) was used to assess respondents' anxiety levels. The scores are summative (as opposed to averaged), which covers all symptoms listed in the DSM-IV^24^. A four-point scale is used to rate GAD-7 responses^24^, with scores indicating levels of anxiety: 5 (mild), 10 (moderate), and 15 (severe). A score of 10 or higher is considered a reasonable threshold for identifying GAD-7^24^. Cronbach's alpha coefficient for GAD-7 in this study was 0.914, showing high reliability.

In the survey, respondents were instructed to choose answers based on the following original prompts: (1) How concerned are you about pandemics and viruses, such as COVID-19 and the H1N1 virus? (2) To what extent are you worried about being infected by COVID-19, H1N1, and other viruses? (3) To what extent are you worried about being unable to work and live normally due to COVID-19, H1N1, and other pandemics? (4) How did COVID-19, H1N1, and other pandemics and viruses impact your health? (5) How did COVID-19, H1N1, and other pandemics and viruses impact your quality of life? (6) How did pandemics and viruses, such as COVID-19 and H1N1, impact your mental health? (7) Are you satisfied with your physical health? (8) Are you satisfied with your quality of life? (9) Are you satisfied with your mental health? We scored responses with a Likert scale of five points. The development process of the nine questions included a comprehensive review of the relevant literature, consultation with experts in mental health research, and consideration of key themes that emerged from pilot surveys with small samples of participants.

Additionally, sociodemographic data was collected on respondents, including gender, age, education status, and marital status.

### Analysis

In this study, SPSS 25.0 was used to compare and analyze the data. The characteristics of the study population were determined with descriptive statistics. Our study examined the relationship between depression, anxiety, and demographic variables by using Chi-square tests. ANOVA was used to examine differences in participants' attitude variables. Variable correlation was assessed using Spearman's correlation. The potential factors of depression and anxiety was determined by an ordered logit regression model. A combination of the results from this study is given as odds ratios (ORs) and 95% confidence intervals (CIs). In this study, *p* < 0.05 was considered statistically significant.

### Sampling procedure and sample size estimation

In our study, we carefully selected participants using a thorough stratified random sampling method, considering gender, age, education, and regional distribution (administrative divisions). This ensured a diverse and representative sample for a comprehensive examination of the target population. We divided the population into strata based on demographics and geographical regions, using random sampling to select individuals in each stratum. To enhance randomness, we added a layer of complete random sampling, selecting respondents within each regional stratum. This meticulous approach addresses the complex interplay between demographic variables and regional differences, allowing nuanced analysis within specific groups and ensuring findings are applicable across diverse geographical regions.

Population Size—According to public data from the Anyang City People’s Government, the resident population of Anyang City was 5,423,000 in 2021^25^. The population size is very large.

Margin of error (confidence interval)—Error is inevitable, so choose a margin of error. Using confidence intervals, we can let our sample mean fall into a higher or lower range than the population mean. Proposition Z was adopted with a 5% margin of error^26^.

Confidence Level—Confidence intervals of 90%, 95%, and 99% are the most common. This study assumed 95% confidence level^26^.

Standard of Deviation—The value of 0.5 is considered to be the most forgiving and it guarantees a large sample size^26^.

The Z-score represents the constant value needed to calculate the confidence level, such as 1.645 for 90%, 1.96 for 95%, and 2.326 for 100%^26^.

So, sample size = (Z-score)^2^ × StdDev × (1-StdDev) / (margin of error)^2^ (CI = 95%, standard deviation = 0.5, margin of error =  + /- 5%) ^26^.

 = {(1.96) ^2^ x 0.5 (0.5)}/(0.05) ^2^.

 = (3.8416 x 0.25) / 0.0025.

 = 0.9604 / 0.0025.

 = 384.16.

385 respondents are needed.

### Ethics

Universiti Sains Malaysia's Human Ethics Committee approved this study (JEPeM Code: USM/JEPeM/22,120,811). Online informed consent was obtained from all the participants. All surveys were anonymous, and each participant completed the same set of questionnaires. This study was based on the tenets of the Declaration of Helsinki.

## Results

This study collected a total of 385 questionnaires. 385 respondents were gender-balanced, with 211 males (54.8%) and 174 females (45.2%). Most respondents were unmarried (n = 279; 72.5%), and 44.7% were between 18 and 24 years old. Over 50% of the participants possessed undergraduate degrees, and 27% had completed junior colleges. Table 1 presents the respondents’ demographic characteristics and reported symptoms related to mental health. Among the respondents, 257 (66.7%) had no or mild depression, while the remaining 128 (33.3%) reported moderate, moderately severe, or severe depression. In addition, 302 (78.5%) respondents exhibited no or mild anxiety, while 83 (21.6%) had moderate or severe anxiety.

**Table 1:** Participant characteristics.

Characteristic	N	Percentage (%)
Gender		
Male	211	54.8
Female	174	45.2
Age		
18–24	172	44.7
25–29	163	42.3
30–34	44	11.4
35–39	6	1.6
Education status		
Primary and below	4	1.0
Junior high school	11	2.9
High school/technical school	46	11.9
Junior college	104	27.0
Bachelor’s degree	215	55.8
Master’s degree and above	5	1.3
Marital status		
Married	99	25.7
Unmarried	279	72.5
Separated	5	1.3
Divorced	2	0.5
Depression (PHQ-9)		
None/minimal	153	39.7
Mild	104	27.0
Moderate	78	20.3
Moderately severe	40	10.4
Severe	10	2.6
Anxiety (GAD-7)		
Minimal	182	47.3
Mild	120	31.2
Moderate	60	15.6
Severe	23	6.0

*PHQ-9* patient health questionnaire-9, *GAD-7* generalized anxiety disorder-7.

The Chi-square test (Tables 2, 3) indicates that educational attainment is significantly correlated with depression and anxiety. Gender was significantly associated with anxiety levels. Compared with men, a greater proportion of women exhibited moderate to severe levels of anxiety. According to the ANOVA (Table 4) results, except for one variable called “worried about not being able to work and live normally due to the pandemic”, other epidemic-related variables had a statistically significant relationship with depression levels. Except for the variable “concerned about pandemics and viruses” which was not related anxiety level, other variables relevant to epidemic were significantly associated with anxiety levels.

**Table 2:** Relationship between depression and demographic variables.

Variable	Description	None-minimal	Mild	Moderate	moderately severe	Severe	Chi-square (p-value)
Gender	Male	94 (44.5%)	55 (26.1%)	39 (18.5%)	19 (9.0%)	4 (1.9%)	5.390 (0.252)
	Female	59 (33.9%)	49 (28.2%)	39 (22.4%)	21 (12.1%)	6 (3.4%)	
Age	18–24	69 (40.1%)	48 (27.9%)	27 (15.7%)	23 (13.4%)	5 (2.9%)	17.745 (0.081)
	25–29	71 (43.6%)	38 (23.3%)	37 (22.7%)	14 (8.6%)	3 (1.8%)	
	30–34	9 (20.5%)	17 (38.6%)	13 (29.5%)	3 (6.8%)	2 (4.5%)	
	35–39	4 (66.7%)	1 (16.7%)	1 (16.7%)	0 (0.0%)	0 (0.0%)	
Education status	Primary and below	2 (50.0%)	1 (25.0%)	1 (25.0%)	0 (0.0%)	0 (0.0%)	46.058 (0.000*)
	Junior high school	3 (27.3%)	1 (9.1%)	4 (36.4%)	3 (27.3%)	0 (0.0%)	
	High school/technical school	10 (21.7%)	16 (34.8%)	8 (17.4%)	9 (19.6%)	3 (6.5%)	
	Junior college	26 (25.0%)	30 (28.8%)	29 (27.9%)	16 (15.4%)	3 (2.9%)	
	Bachelor’s degree	108 (50.2%)	55 (25.6%)	36 (16.7%)	12 (5.6%)	4 (1.9%)	
	Master’s degree and above	4 (80.0%)	1 (20.0%)	0 (0.0%)	0 (0.0%)	0 (0.0%)	
Marital status	Married	38 (38.4%)	29 (29.3%)	20 (20.2%)	7 (7.1%)	5 (5.1%)	13.843 (0.249)
	Unmarried	113 (40.5%)	72 (25.8%)	58 (20.8%)	31 (11.1%)	5 (1.8%)	
	Separated	2 (40.0%)	1 (20.0%)	0 (0.0%)	2 (40.0%)	0 (0.0%)	
	Divorced	0 (0.0%)	2 (100.0%)	0 (0.0%)	0 (0.0%)	0 (0.0%)	

*p < 0.05.

**Table 3:** Relationship between anxiety and demographic variables.

Variable	Description	Minimal Anxiety	Mild Anxiety	Moderate Anxiety	Severe Anxiety	Chi-square (*p*-value)
Gender	Male	112 (53.1%)	64 (30.3%)	22 (10.4%)	13 (6.2%)	11.388 (0.009*)
	Female	70 (40.2%)	56 (32.2%)	38 (21.8%)	10 (5.7%)	
Age	18–24	85 (49.4%)	49 (28.5%)	26 (15.1%)	12 (7.0%)	11.260 (0.214)
	25–29	79 (48.5%)	52 (31.9%)	23 (14.1%)	9 (5.5%)	
	30–34	13 (29.5%)	19 (43.2%)	10 (22.7%)	2 (4.5%)	
	35–39	5 (83.3%)	0 (0.0%)	1 (16.7%)	0 (0.0%)	
Education status	Primary and below	2 (50.0%)	2 (50.0%)	0 (0.0%)	0 (0.0%)	48.726 (0.000*)
	Junior high school	5 (45.5%)	0 (0.0%)	3 (27.3%)	3 (27.3%)	
	High school/technical school	18 (39.1%)	17 (37.0%)	6 (13.0%)	5 (10.9%)	
	Junior college	27 (26.0%)	46 (44.2%)	23 (22.1%)	8 (7.7%)	
	Bachelor’s degree	126 (58.6%)	54 (25.1%)	28 (13.0%)	7 (3.3%)	
	Master’s degree and above	4 (80.0%)	1 (20.0%)	0 (0.0%)	0 (0.0%)	
Marital status	Married	45 (45.5%)	31 (31.3%)	17 (17.2%)	6 (6.1%)	9.311 (0.335)
	Unmarried	133 (47.7%)	88 (31.5%)	43 (15.4%)	15 (5.4%)	
	Separated	3 (60.0%)	0 (0.0%)	0 (0.0%)	2 (40.0%)	
	Divorced	1 (50.0%)	1 (50.0%)	0 (0.0%)	0 (0.0%)	

*p < 0.05.

**Table 4:** One-way analysis of variance (ANOVA) results.

	Depression			Anxiety		
	df	F	P	df	F	P
Concerned about pandemics and viruses	4	3.149	0.014*	3	1.269	0.285
Worried about being infected by viruses	4	3.153	0.014*	3	5.916	0.001*
Worried about not being able to work and live normally due to the pandemic	4	2.223	0.066	3	4.008	0.008*
Impact of pandemics and viruses on physical health	4	4.468	0.002*	3	6.438	0.000*
Impact of pandemics and viruses on quality of life	4	4.865	0.001*	3	6.294	0.000*
Impact of pandemics and viruses on mental health	4	3.857	0.004*	3	6.483	0.000*
Satisfaction with physical health	4	29.156	0.000*	3	30.260	0.000*
Satisfaction with quality-of-life situation	4	28.643	0.000*	3	22.446	0.000*
Satisfaction with mental health	4	29.737	0.000*	3	32.548	0.000*

*p < 0.05.

The correlation analysis for depression burden, anxiety burden, and other factors is shown in Table 5. Depression burden was significantly and positively correlated with gender, worry about being infected by viruses, and the impact of pandemics and viruses on physical health, quality-of-life, and mental health (i.e., 3, 8, and 10–12 in Table 5). It was also significantly and negatively correlated with education status, concern about pandemics and viruses, and satisfaction with physical health, quality-of-life situation, and mental health (i.e., 5, 7, and 13–15 in Table 5). Meanwhile, anxiety burden was significantly and positively correlated with gender, worry about being infected by viruses and not being able to work and live normally due to the pandemic, and the impact of pandemics and viruses on physical health, quality-of-life, and mental health (i.e., 2 and 8–12 in Table 5). Anxiety burden was significantly and negatively correlated with education status, concern about pandemics and viruses, and satisfaction with physical health, quality-of-life situation, and mental health (i.e., 5, 7, and 13–15 in Table 5).

**Table 5:** Spearman’s correlation between anxiety, depression burden, and factors.

	Depression (PHQ− 9)	Anxiety (GAD− 7)
(1) Depression (PHQ-9)	1	0.795**
(2) Anxiety (GAD-7)	0.795**	1
(3) Gender	0.115*	0.141**
(4) Age	0.021	0.040
(5) Education status	− 0.289**	− 0.247**
(6) Marital status	0	− 0.023
(7) Concerned about pandemics and viruses	− 0.195**	− 0.119*
(8) Worried about being infected by viruses	0.104*	0.150**
(9) Worried about not being able to work and live normally due to the pandemic	0.098	0.156**
(10) Impact of pandemics and viruses on physical health	0.191**	0.213**
(11) Impact of pandemics and viruses on quality of life	0.187**	0.210**
(12) Impact of pandemics and viruses on mental health	0.181**	0.213**
(13) Satisfaction with physical health	− 0.455**	− 0.420**
(14) Satisfaction with quality-of-life situation	− 0.456**	− 0.362**
(15) Satisfaction with mental health	− 0.469**	− 0.429**

*p < 0.05, **p < 0.01.

Table 6 presents the ordered logit regression results for depression. According to the results, respondents who were less concerned about pandemics and viruses, whose mental health was more impacted by pandemics and viruses, and displayed lower contentment with both their physical and mental health, exhibited a heightened susceptibility to depression.

**Table 6:** Ordered logit regression analysis of factors influencing depression.

Variable	B	P	OR	95% CI	
				Lower	Upper
Male	− 0.246	0.222	0.782	0.526	1.161
Female	0^a^				
18–24	1.167	0.222	3.213	0.494	20.880
25–29	0.822	0.383	2.274	0.359	14.422
30–34	0.395	0.682	1.485	0.224	9.834
35–39	0^a^				
Primary and below	1.018	0.510	2.768	0.134	57.280
Junior high school	2.325	0.078	10.223	0.770	135.817
High school/technical school	1.921	0.114	6.827	0.631	73.862
Junior college	1.636	0.170	5.135	0.495	53.254
Bachelor’s degree	1.098	0.353	2.999	0.296	30.412
Master’s degree and above	0^a^				
Married	0.326	0.814	1.386	0.092	20.971
Unmarried	0.112	0.935	1.119	0.076	16.542
Separated	0.585	0.717	1.795	0.076	42.221
Divorced	0^a^				
Concerned about pandemics and viruses	− 0.250	0.044*	0.779	0.611	0.993
Worried about being infected by viruses	− 0.105	0.454	0.901	0.685	1.184
Worried about not being able to work and live normally due to the pandemic	0.061	0.664	1.063	0.807	1.400
Impact of pandemics and viruses on physical health	0.111	0.415	1.117	0.856	1.459
Impact of pandemics and viruses on quality of life	0.010	0.943	1.010	0.765	1.334
Impact of pandemics and viruses on mental health	0.320	0.018*	1.377	1.057	1.793
Satisfaction with physical health	− 0.298	0.039*	0.742	0.560	0.985
Satisfaction with quality-of-life situation	− 0.180	0.223	0.835	0.626	1.116
Satisfaction with mental health	− 0.297	0.033*	0.743	0.565	0.977

*ORs* odds ratios, *CI* confidence intervals.

*p < 0.05.

As shown in Table 7, anxiety was analyzed using an ordered logit regression. Results indicate that respondents whose mental health was more impacted by pandemics and viruses and who were less satisfied with their physical and mental health were at greater risk of suffering from anxiety. Moreover, respondents with a junior high school education level exhibited more anxiety burden than those with master’s degrees or higher.

**Table 7:** Ordered logit regression analysis of factors influencing anxiety.

Variable	B	P	OR	95% CI	
				Lower	Upper
Male	− 0.356	0.089	0.700	0.464	1.056
Female	0^a^				
18–24	1.769	0.106	5.867	0.685	50.258
25–29	1.501	0.167	4.486	0.534	37.663
30–34	1.018	0.357	2.768	0.317	24.179
35–39	0^a^				
Primary and below	1.027	0.515	2.792	0.127	61.502
Junior high school	2.662	0.044*	14.320	1.070	191.611
High school/technical school	1.162	0.338	3.198	0.296	34.556
Junior college	1.613	0.175	5.018	0.487	51.686
Bachelor’s degree	0.854	0.469	2.348	0.233	23.643
Master's degree and above	0^a^				
Married	1.187	0.475	3.277	0.127	84.835
Unmarried	0.905	0.583	2.472	0.097	62.750
Separated	1.404	0.451	4.071	0.105	157.170
Divorced	0^a^				
Concerned about pandemics and viruses	− 0.110	0.402	0.896	0.693	1.159
Worried about being infected by viruses	− 0.105	0.466	0.900	0.678	1.195
Worried about not being able to work and live normally due to the pandemic	0.158	0.278	1.171	0.880	1.559
Impact of pandemics and viruses on physical health	0.130	0.356	1.139	0.864	1.503
Impact of pandemics and viruses on quality of life	− 0.058	0.694	0.944	0.706	1.260
Impact of pandemics and viruses on mental health	0.288	0.038*	1.334	1.016	1.752
Satisfaction with physical health	− 0.344	0.021*	0.709	0.528	0.950
Satisfaction with quality-of-life situation	0.055	0.719	1.056	0.784	1.424
Satisfaction with mental health	− 0.456	0.002*	0.634	0.476	0.842

*ORs* dds ratios, *CI* confidence intervals.

*p < 0.05.

## Discussion

Among young adults in Anyang, China, we examined self-reported depression and anxiety burden and factors associated with these symptoms following the COVID-19 pandemic and resurgence of H1N1 influenza. Results can be summarized as follows.

The results showed that 33.3% of respondents scored 10 or higher on the PHQ-9 when it came to depression, and 21.6% on the GAD-7 when it came to anxiety. There was a marked increase in depression and anxiety rates compared with previous studies conducted in China. For instance, from September to November 2019, a population-based study found that 8.6% of Guangdong residents suffer from depression and 6% anxiety^27^. Studying Hong Kong's general population from April 24 to May 3, 2020, 14% of the participants exhibited symptoms indicative of anxiety burden, and 19% displayed signs of depression burden^28^. In a post-epidemic study in September 2020, 25.9% of young people in Wuhan, Hubei Province, the epicenter of the epidemic, reported depressive symptoms^29^. The prevalence of anxiety in the population of Beijing during COVID-19's second wave was 15.3% from June 22, 2020 to June 26, 2020^30^. Young adults in Anyang may have experienced mood disorders during the renewed H1N1 outbreak in February 2023, following the COVID-19 period. According to previous studies, H1N1 influenza caused great psychological distress to young people in China^31^. Susceptibility, severity, and perceptions associated with community outbreaks are all related to the mental health conditions caused by H1N1 influenza^17^. In addition, the impact of H1N1 influenza on young people is manifested by mental distress/mood disorders that produce worry, fear, depression, and emotional upset^17^. Therefore, young adults in Anyang City have yet to regain mental health normality following the COVID-19 pandemic and H1N1 influenza.

The results of this study confirm several findings of previous studies. More specifically, depression and anxiety levels positively correlated with the effects of the COVID-19 pandemic and the H1N1 influenza on mental health, physical health, and quality of life. These findings align with prior studies, including Hao et al.^32^ recent study of COVID-19 infected patients in psychiatric care, which found that poorer physical health contributed to both worse anxiety and depression. Additionally, individuals with lower levels of physical activity before and during COVID-19 experienced worsened mental health, including increased anxiety and depression as well as decreased life satisfaction^33^. This study found a positive correlation between anxiety levels and worry about not being able to work and live a normal life due to the pandemic. This finding aligns with a 2020 US study that linked increased job insecurity, financial concerns, worries about COVID-19, concerns about the effects on daily life caused by the pandemic, and higher risk of infection to more severe anxiety symptom^34^.

Results also showed that satisfaction with physical health, life quality, and mental health had an inverse correlation with anxiety and depression levels during pandemics and outbreaks. This finding aligns with those of previous research. In 2009, the WHO reported that global uncertainty regarding the safety of pandemic influenza testing and decreased satisfaction with health information were associated with increased pandemic-related psychological distress, stress, anxiety, and depression^35^. Based on the research conducted by Gierc et al., exercise amidst the COVID-19 pandemic exhibited a connection with diminished occurrences of depression and anxiety, alongside a rise in overall life contentment^33^. In addition, COVID-19 fear has been demonstrated to increase depression, anxiety, and stress while decreasing life satisfaction^36^. The study found a negative correlation between educational status and depression and anxiety levels. These results are also in agreement with prior studies, including a 2008 Norwegian study which found that low levels of education were related to anxiety and depression^37^.

Participants' concerns about pandemics and viruses had a significant negative impact relationship on depression levels in this study. This finding is consistent with previous research. For example, Chang et al. (2020) showed that outbreak information overload not only triggers worry, but also promotes increased awareness of the outbreak; the greater the understanding of the COVID-19 situation, the more likely hygiene habits in the future will be altered, resulting in reduced anxiety and depression levels among young college students^38^. In addition to providing a better understanding of the epidemic and prevention measures, a psychologically healthier college student will be able to deal with the situation more effectively^38^. In the post-pandemic period, economic prospects, job security, and finding work may be potential reasons for greater mood disorders during this period, especially since these age groups are in their prime employment years. Mental distress appears to be influenced by the employment situation, financial instability, and inadequate income, while advancing age emerges as a safeguard against it^39^. Therefore, young people in Anyang should pay more attention to the health education knowledge on epidemic prevention and control measures provided by relevant government departments on various online platforms.

Furthermore, consistent with previous research, this study found that younger adults with a junior high school education may be more prone to experiencing anxiety symptoms than those with a master’s degree or higher. For instance, Melkevik et al. found that lower educational attainment was correlated with an increased risk of anxiety symptoms^40^. Yang et al. discovered a positive correlation between higher educational levels among Chinese individuals and increased happiness, as well as between educational level and income^41^. People with a junior high school education typically have inferior employment and career development opportunities compared to those with master's degrees or higher. Therefore, young adults with junior high school educations may feel more uncertain about their financial and career prospects than those with a higher education level.

We recognize some limitations associated with the use of self-reported data. Firstly, the study simply uses the population size of administrative divisions as the basis for stratified sampling. We acknowledge that the urban–rural divide is a crucial factor influencing mental health outcomes. The failure to explicitly consider residence during stratification limits the depth of our analysis and may obscure important variations in depression and anxiety levels among different populations. To address this limitation, future research endeavors should incorporate residence as a key stratification variable. In this way, mental health dynamics in the Anyang region can be better understood across urban and rural areas. Secondly, the limitation of not factoring prevalence into the sample size calculation underscores the need for cautious interpretation of findings. While our study provides valuable insights into mental health within the younger population, caution should be exercised when extrapolating these findings to the broader population, as the sample size was optimized for our specific exploratory objectives. Furthermore, our results might be limited in generalizability, as they are specific to the time and population under examination. Different contexts and timeframes could yield varying results, emphasizing the need for caution when extrapolating our findings. Finally, while online surveys offer advantages in terms of broad reach, we acknowledge potential limitations related to digital accessibility, variations in internet connectivity, and disparities in technology access. Future research could explore strategies to enhance inclusivity in online mental health surveys.

This study has several important implications. In a 28-month study, it was shown that anxiety-type psychological responses were reduced by about 50% over the 28-month period (in the absence of another public health crisis)^42^. In contrast, this study indicates that anxiety/depression prevalence remained high over time in the face of another (H1N1) virus outbreak immediately after the COVID-19 pandemic. This study deals with recurring epidemics. Another historical study has shown that pandemic patients (SARS) suffered a long-term mental health impact (lasting 1 year)^43^. A long-term COVID-19 outbreak could also worsen the mental health effects of another disease outbreak (H1N1) in the community. Government agencies can also benefit from this study's findings when developing policies aimed at improving young adults' mental health. In this respect, the government and relevant departments, such as hospitals, should provide integrated health services to improve the health system’s resilience during health emergencies^44^. Cities also need to focus on young people to prevent further negative effects in post-pandemic periods. The harmonization of work and life and regulation of physical and mental health are especially important for controlling depression and anxiety.

## Conclusion

The study found that mental health of females and those with low levels of education may be more affected by successive and concurrent epidemics. Moreover, pandemics and viruses were positively correlated with the participants' anxiety and depression levels. The level of contentment pertaining to their physical and mental health demonstrated an inverse correlation with both anxiety and depression. Furthermore, participants' educational status significantly positively influenced anxiety levels; participants' concern about pandemics and viruses had a significant negative impact on depression levels. Government departments, scholars, and clinical psychologists should focus on young adults’ mental health to mitigate the harm caused by pandemics and health crises. Moreover, young adults should use various strategies to alleviate depression and anxiety burden, such as engaging in physical exercise and finding suitable distractions.

## Data Availability

In view of ethical considerations, the information produced and/or assessed throughout this research cannot be disclosed to the public; however, interested individuals may request the data from the corresponding author through a reasonable inquiry.
